# Mechanisms of ferroptosis in chronic kidney disease

**DOI:** 10.3389/fmolb.2022.975582

**Published:** 2022-08-24

**Authors:** Wen-Qing Zhuo, Yi Wen, Hui-Jun Luo, Zhu-Lin Luo, Li Wang

**Affiliations:** ^1^ Department of Nephrology, Sichuan Provincial People’s Hospital, University of Electronic Science and Technology of China, Chengdu, China; ^2^ Chinese Academy of Sciences Sichuan Translational Medicine Research Hospital, Chengdu, China; ^3^ Department of General Surgery and Pancreatic Injury and Repair Key Laboratory of Sichuan Province, The General Hospital of Western Theater Command (Chengdu Military General Hospital), Chengdu, Sichuan, China; ^4^ Department of Biochemistry and Molecular Biology, Mayo Clinic Arizona, Scottsdale, AZ, United States

**Keywords:** ferroptosis, chronic kidney disease (CKD), regulatory genes, review, therapeutic strategy

## Abstract

Ferroptosis is a newly identified form of regulated cell death characterized by iron accumulation and lipid peroxidation. Ferroptosis plays an essential role in the pathology of numerous diseases and has emerged as a key area of focus in studies of chronic kidney disease (CKD). CKD is a major public health problem with high incidence and mortality that is characterized by a gradual loss of kidney function over time. The severity and complexity of CKD combined with the limited knowledge of its underlying molecular mechanism(s) have led to increased interest in this disease area. Here, we summarize recent advances in our understanding of the regulatory mechanism(s) of ferroptosis and highlight recent studies describing its role in the pathogenesis and progression of CKD. We further discuss the potential therapeutic benefits of targeting ferroptosis for the treatment of CKD and the major hurdles to overcome for the translation of *in vitro* studies into the clinic.

## Introduction

Ferroptosis is an iron-dependent form of non-apoptotic cell death, first described in 2012 by Dixon and colleagues (Dixon et al., 2012). This form of regulated necrosis is characterized by lipid peroxidation at the plasma membrane and subcellular components, ultimately leading to cell rupture (Dixon et al., 2012; Yang and Sockwell, 2016; Latunde-Dada, 2017; Galluzzi et al., 2018; Gao et al., 2019; Hirayama et al., 2019; Li et al., 2020). Ferroptosis has distinct morphological and biochemical features that distinguishes it from other forms of cell death. Morphologically, ferroptosis is characterized by unique mitochondrial changes, including the rupture of mitochondria membranes, a reduction in mitochondrial crests and a decrease in mitochondria numbers (Otasevic et al., 2021). The contribution of mitochondria to ferroptosis has emerged as a promising target to prevent cell death through blocking ferroptosis. Biochemically, ferroptosis is characterized by an increased consumption of glutathione (GSH) and decreased activity of glutathione peroxidase 4 (GPX4) and system Xc^−^ (a cysteine/glutamate antiporter system). Other biochemical features of ferroptosis include an increased production of reactive oxygen species (ROS), an accumulation of lipid peroxides and aberrant iron metabolism (Zheng and Conrad, 2020; Chen et al., 2021). In recent years, ferroptosis has emerged as a critical contributor and potential therapeutic target in the context of a range of pathologic states, including acute and chronic kidney disease, cardiovascular and neurodegenerative disease, stroke and chemotherapy-resistant cancers, amongst others (Xie et al., 2016; Stockwell et al., 2017; Conrad et al., 2021; Maremonti et al., 2022).

Chronic kidney disease (CKD) is a major global public health problem that afflicts 8%–16% of the population and is estimated to contribute to 5–10 million deaths annually (WHO, 2018; Xie et al., 2018; Chen et al., 2019). CKD is characterized by kidney damage (albuminuria) and a gradual decline in kidney function (estimated glomerular filtration rate (eGFR) < 60 ml/min/1.73 m^2^) (Levey et al., 2005). Cardiovascular disease is a significant adverse outcome in CKD patients that increases its mortality rates (Chandrajay, 2010). Despite improvements in the care of CKD patients and disease management, life expectancy remains low across all stages (Turin et al., 2012) and its global burden continues to rise (Xie et al., 2018). A deeper understanding of the molecular mechanism(s) governing CKD pathogenesis are required to delay its progression, improve diagnostics and to advance the discovery of novel therapeutics.

In recent years, ferroptosis has been widely studied in the context of kidney disease (Müller et al., 2017; Wenzel et al., 2017; Guan et al., 2021; Li et al., 2021) and has emerged as a major area of focus in CKD studies. Understanding the regulation of ferroptosis in CKD is a pre-requisite to reduce kidney cell death and its associated morbidity and mortality.

Herein, we review our latest understanding of ferroptosis in the kidneys and discuss its regulatory genes and links to CKD. We further describe challenges and future perspectives for the use of modulators of ferroptosis as much needed anti-CKD therapeutics.

## General mechanisms of ferroptosis

**FIGURE 1:**
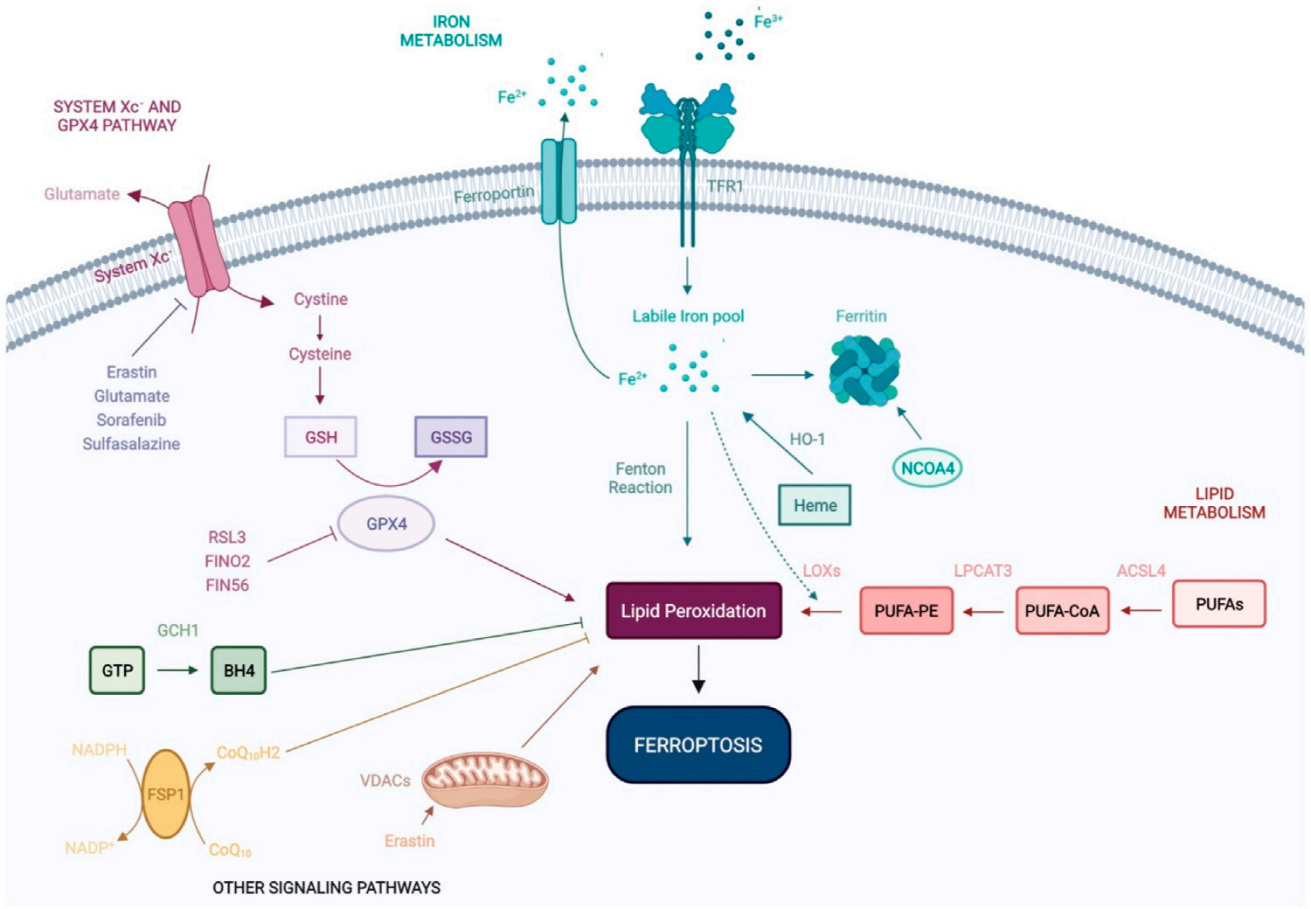
Molecular mechanisms of ferroptosis. Ferroptosis is an iron-dependent form of non-apoptotic cell death, regulated by a range of molecular mechanisms and metabolic pathways, including system Xc^−^ and GPX4 signaling, and iron and lipid metabolism. Recent studies have shown that VDACs and other signaling pathways—FSP1-CoQ10-NADPH and GCH1-BH4 work cooperatively with the GPX4/glutathione system to regulate lipid peroxidation and ferroptosis. GSH: Glutathione; GSSG: Glutathione disulfide; GPX4: Glutathione Peroxidase 4; Fe^2+^: Ferrous iron; Fe^3+^: Ferric iron; TFR1: Transferrin Receptor 1; HO-1: Heme oxygenase-1; NCOA4: Nuclear receptor coactivator 4; LOXs: Lipoxygenases; LPCAT3: lysophosphatidylcholine acyltransferase 3; ACSL4: Acyl-CoA Synthetase Long Chain Family Member 4; PUFAs: Polyunsaturated fatty acids; PUFA-CoA: Polyunsaturated fatty acyl-coenzyme A; PUFA-PE: Polyunsaturated fatty acid-phosphatidylethanolamine; GTP: Guanosine triphosphate; BH4: tetrahydrobiopterin; GCH1: Guanosine triphosphate cyclohydrolase 1; FSP1: Ferroptosis suppressor protein 1; VDACs: Voltage-dependent anion channels; CoQ10: Coenzyme Q10.

Ferroptosis is regulated by a multitude of metabolic and signaling pathways, including system Xc^−^ (Cysteine/Glutamate Antiporter), GPX4, iron homeostasis, ROS and lipid signaling. In this section, we summarize the role of these pathways during ferroptosis (Figure 1).

## System Xc^−^ and glutathione peroxidase 4 pathways

System Xc^−^ is a widely occurring cysteine (Cys)/glutamate (Glu) antiporter, first identified by Banai et al. (1986) and a known regulator of ferroptosis (Badgley et al., 1979) System Xc is composed of light and heavy chains that are encoded by *SLC7A11* and *SLC3A2*, respectively (Lewerenz et al., 2013). System Xc^−^ mediates the exchange of Glu and Cys across the plasma membrane, ultimately favoring reduced glutathione (GSH) synthesis (Koppula et al., 2018; Liu et al., 2021). Inhibition of system Xc^−^ decreases GSH expression, leading to oxidative damage and ferroptosis. Erastin (the first identified inducer of ferroptosis), extracellular glutamate, sorafenib and sulfasalazine, can block system Xc^−^ and trigger ferroptosis (Dixon et al., 2012; Gao et al., 2015; Zhao et al., 2020).

GPX4 is a peroxide-degrading enzyme that uses GSH as a substrate to produce glutathione disulfide (GSSG), ultimately preventing lipid peroxidation and maintaining redox homeostasis (Ingold et al., 2018). The genetic ablation of GPX4 (Seiler et al., 2008) or its pharmacological inhibition with RSL3 (Yang and Stockwell, 2008) leads to impaired antioxidant capacity and favors cell death by ferroptosis, independently of system Xc status (Yang et al., 2014). In addition to its ability to directly inactivate GPX4, erastin indirectly suppresses GPX4 through the upregulation of activating transcription factor 3 (ATF3) (Zhang et al., 2021). FINO2 and FIN56 have also been identified as direct- and indirect inhibitors of GPX4 activity, respectively (Gaschler et al., 2018).

## Iron metabolism

Iron overload is one of the major hallmarks of ferroptosis. Iron is naturally present in the human body, however its active redox activity favors ROS production and lipid peroxidation, ultimately leading to ferroptosis (Hassannia et al., 2019; Battaglia et al., 2020; Li et al., 2020). Under physiological conditions, iron uptake and export are regulated by transferrin receptor and ferroportin respectively at the extracellular level, and by ferritin (iron storage) intracellularly. Ferritin is an intracellular protein that oxidizes ferrous iron (Fe^2+^) to ferric iron (Fe^3+^) avoiding the occurrence of the Fenton reaction and subsequent oxidative damage (Hassannia et al., 2019). Ferritin promotes iron storage, its degradation leading to the release of iron stores and subsequent ferroptosis (Rui et al., 2021). Controlling iron metabolism/homeostasis holds great potential for the control of ferroptosis. Accordingly, it has been shown that iron homeostasis is related to the nuclear receptor activator 4 (NCOA4), which can mediate ferritinophagy, a process in which ferritin is delivered to lysosomes and is selectively degraded *via* autophagy (Mancias et al., 2014; Gao et al., 2016; Hou et al., 2016; Torii et al., 2016; Buccarelli et al., 2018). Heme oxygenase-1 (HO-1), catalyzes the degradation of heme to produce Fe^2+^ and contributes to iron homeostasis (Chang et al., 2018; Tang et al., 2018). The activation of ferritinophagy and/or HO-1 overexpression increases free iron levels, leading to the accumulation of lipid peroxides and subsequent ferroptosis.

## Lipid metabolism

Lipid metabolism is closely associated with ferroptosis and can be triggered by non-enzymatic (Fenton chemistry) and enzymatic mechanisms [lipoxygenases (LOXs)] (Doll and Conrad, 2017; Feng and Stockwell, 2018). Polyunstaturated fatty acids (PUFAs), such as arachidonic acid (AA) and adrenoyl acid are phospholipids that increase the susceptibility to lipid peroxidation during ferroptosis (Friedmann Angeli et al., 2014; Kagan et al., 2017; Wenzel et al., 2017), their abundance and localization impacting lipid peroxidation and the sensitivity of cells to ferroptosis (Doll and Conrad, 2017; Stockwell et al., 2017). More specifically, PUFA metabolism by Fe^2+^ and LOXs leads to the production of lipid peroxides that disturb membrane structure and function (Hassannia et al., 2019). In this regard, the fatty acid metabolism gene *Acyl-CoA synthetase long-chain family member 4 (ASCL4)* and lipid remodeling gene *Lysophosphatidylcholine acyltransferase 3 (LPCAT3)*, regulate the insertion of PUFAs into cellular membranes and have been identified as pivotal biomarkers of ferroptosis (Dixon et al., 2015). ACSL4 silencing inhibits ferroptosis, whilst its overexpression modulates cellular lipid composition and sensitivity to ferroptosis (Yuan et al., 2016; Doll et al., 2017). Increasing the expression and/or catalytic activity of ASCL4, LPCAT3, and LOXs or the Fenton reaction increases the accumulation of lipid peroxides and ultimately, ferroptosis (Li and Li, 2020).

## Other signaling pathways

Recent studies have revealed a range of signaling pathways that regulate ferroptosis in a multitude of cellular systems. The mevalonate pathway has been shown to inhibit ferroptosis in studies by Stockwell et al. (2017), and two independent studies identified *FSP1* [ferroptosis suppressor protein 1, formerly named Gene apoptosis-inducing factor mitochondrial 2 (AIFM2)], as a novel ferroptosis resistance gene. *FSP1* was shown to complement the loss (GPX4 KO) or inhibition (RLS3-treated) of the master regulator GPX4 (Bersuker et al., 2019; Doll et al., 2019). FSP1 NADH-dependent oxireductase is recruited to membranes of organelles where it converts oxidase CoQ10 (ubiquinone) into reduced CoQ10 (ubiquinol), preventing oxidative lipid production and its incorporation into membranes and lipoproteins. Collectively, this highlights new methods to suppress ferroptosis independently of GPX4.

In recent studies by Kraft et al. (2020), Guanosine triphosphate (GTP) cyclohydrolase 1 (GCH1), the rate-limiting enzyme in the synthesis of the antioxidant tetrahydrobiopterin (BH4), was identified as a highly potent suppressor of ferroptosis. More specifically, the overexpression of GCH1 favored BH4 synthesis, suppressing ferroptosis by abolishing lipid peroxidation. The GCH1-BH4 axis is a master regulator of ferroptosis resistance, controlling the endogenous production of BH4, the abundance of CoQ10, and the peroxidation of phospholipids independently of the GPX4/glutathione system.

Mitochondria drive the production of cellular ROS and play a central role in ferroptosis by promoting lipid peroxidation. Voltage-dependent anion channels (VDACs) transport iron and metabolites and are essential to ferroptosis (DeHart et al., 2018). Erastin inhibits VDACs, leading to mitochondrial dysfunction, ROS production and iron-mediated cell death (Yagoda et al., 2007).

## Ferroptosis and chronic kidney disease

CKD is a major public health issue (WHO, 2018; Xie et al., 2018; Chen et al., 2019). The two main risk factors contributing to the increasing prevalence of CKD are diabetes and hypertension. Other causes include primary glomerulonephritis, inherited diseases such as polycystic kidney disease, kidney stones and repeated urinary infections (Couser et al., 2011). Despite recent interest into the molecular mechanisms regulating CKD pathogenesis and the contribution of environmental and genetic risk factors to disease susceptibility and heterogeneity (Cañadas-Garre et al., 2019), considerable knowledge gaps remain. As a consequence, therapeutic approaches for the management of CKD progression remain scarce.

The kidney is an iron metabolism-related organ. A loss of GPX4 activity (Friedmann Angeli et al., 2014), through genetic deletion leads to albuminuria, kidney tubular epithelial cell death and mortality within weeks (Friedmann Angeli et al., 2014). More specifically in the context of CKD, preclinical studies support the correlation between renal iron deposition, lipid deposition and ferroptosis in multiple forms of CKD, highlighting its clinical significance (Nankivell et al., 1992; Wang et al., 2001; Shah et al., 2007; Martines et al., 2013; Wenzel et al., 2017; Guan et al., 2021; Li et al., 2021). Improved understanding of the mechanisms regulating ferroptosis in the kidney and defining key genes regulating these processes will advance the discovery of new molecular targets for multiple forms of kidney disease.

## Ferroptosis regulatory genes in chronic kidney disease

Ferroptosis regulates CKD and kidney function. The altered expression of genes that regulate ferroptosis may influence the incidence and predisposition to CKD. Table 1 summaries recent advances in this area.

**TABLE 1:** Expression of ferroptosis-regulatory genes in CKD models.

CKD model	Genes	Outcome	References
Kidney biopsy tissue	*Slc7a11 Gpx4*	Decreased expression compared to non-DN conditions	Kim et al. (2021)
TGF-β1-exposed proximal tubular epithelial cells (NRK-52E cells)			
STZ-induced DN mice			
STZ-induced DN and db/db mice	*Acsl4*	Increased expression	Wang et al. (2020)
	*Gpx4*	Decreased expression	
STZ-induced DN rat and immortalized mouse podocytes	*Ho-1*	Increased expression of HO-1 prevents podocyte apoptosis in diabetic models	Lee et al. (2009)
DN patients	*Ferritin*	Increased expression	Wu et al. (2021)
	*Ldh*		
	*ROS*		
	*Mda*		
	*Hmgb1*		
	*Acsl4*		
	*Ptgs2*		
	*Nox1*		
	*Gpx4*	Decreased expression	
Mesangial cells	*Hmgb1*	Increased HMGB1 expression regulates glucose-induced ferroptosis *via* Nrf2	Wu et al. (2021)
Doxorubicin induced-renal fibrosis	*Ptgs2*	Increased mRNA levels	Fang et al. (2019)
UUO or IRI-induced fibrosis mouse kidneys	*Gpx4*	Decreased expression	Li et al. (2017); Zhou et al. (2022)
	*4-HNE*	Increased expression	
Pdk1 mutant renal epithelial cells and Pkd1^RC/RC^ mice	*GSH*	Decreased expression	Zhang et al. (2021)
	*Ferroportin*		
	*Gpx4*		
	*Tfr1*	Increased expression	
	*Dmt1*		
	*Ho-1*		
	*4-HNE*	Increased expression in *Pkd1* null cells, promotes cell proliferation *via* activation of Akt, S6, Stat3 and Rb	

TGF-β1, Transforming growth factor 1; STZ, streptozotocin; DN, diabetic nephropathy; UUO, unilateral ureter obstruction; IRI, Ischemia/reperfusion injury; Pkd1, Polycystin-1; SLC7A11, Solute Carrier Family seven Member 11; GPX4, Glutathione peroxidase 4; ACSL4, Acyl-CoA, Synthetase Long Chain Family Member 4; HO-1, Heme oxygenase-1; LDH, lactate dehydrogenase; ROS, reactive oxygen species; MDA, Melanoma differentiation-associated gene; HMGB1, High Mobility Group Box one; PTGS2, Prostaglandin-Endoperoxide Synthase; NOX1, NADPH, Oxidase 1; 4-HNE, 4-Hydroxynonenal; GSH, gluthatione; TFR1, Transferrin receptor protein one; DMT1, Divalent metal transporter one; Nrf2, Nuclear factor-erythroid factor 2-related factor 2.

To date, approximately 250 kidney function-associated *loci* have been identified, with around 50% showing relevance to kidney function in the context of CKD (Wuttke et al., 2019). Despite promise in this area, only a small number of these genes have been functionally characterized including *UMOD* (Trudu et al., 2013)*, DAB2* (Qiu et al., 2018), *SHROOM3* (Miao et al., 2021), *DACH1* (Doke et al., 2021) and *MANBA* (Gu et al., 2021)*.* In recent genome-wide association studies for kidney disease, *Dpep1* and *Chmp1a*, were identified as key regulators of ferroptosis (Guan et al., 2021). At the molecular level, *Dpep1* and *Chmp1* were shown to alter cellular iron trafficking, ultimately favoring the development of kidney disease. To our knowledge, this is the only study to-date that identifies two potential casual genes of CKD progression that are important regulators of ferroptosis. This highlights the potential therapeutic benefits of pharmacologically targeting ferroptosis through DPEP1 and/or CHMP1 in patients with kidney disease to prevent multiple forms of CKD.

Other ferroptosis regulatory genes that are not specifically identified as casual genes of CKD progression have been studied in the context of diabetic nephropathy (DN), renal fibrosis and autosomal dominant polycystic kidney disease (ADPKD). These are summarized in Table 1.

DN is the most common cause of mortality and morbidity in CKD patients (Iyengar et al., 2015). The main factors involved in DN pathogenesis include high glucose levels, oxidative stress and inflammatory responses (Perrone et al., 2021). Recent studies suggest that, in addition to other forms of programmed cell death, ferroptosis plays a crucial pathological role in the development of DN (Wang et al., 2020). In studies using *in vitro* (proximal kidney tubular cells), *in vivo* [streptozotocin (STZ)-induced DN mice model] and *ex vivo* (kidney biopsy samples) models, a significant reduction in the mRNA and protein expression of the ferroptosis-related molecules SLC7A11 and GPX4 were observed, leading to increased lipid peroxidation in DN compared to non-DN models (Kim et al., 2021). In addition, the changes associated with ferroptosis under diabetic conditions were ameliorated by specific ferroptosis inhibitors. Studies using STZ-induced DN and *db/db* mice further confirmed the involvement of ferroptosis in the progression of DN (Wang et al., 2020). More specifically, significant changes in the expression of the ferroptosis-associated markers ACSL4 and GPX4 were observed. This was accompanied by an increase in lipid peroxidation and iron content in DN mice. *In vitro* studies using known inducers of ferroptosis including erastin and RSL3, induced renal tubular cell death through increasing iron levels and ACSL4 expression, sensitizing cells to ferroptosis (Wang et al., 2020). Other studies showed that the increased expression of HO-1 favors the inhibition of oxidative stress and the restoration of redox balance, which may be beneficial for DN (Lee et al., 2009; Rodriguez et al., 2011). High-mobility box-1 (HMGB1) was shown to be activated in DN patients and mesangial cells in response to high glucose. In studies using high glucose-treated mesangial cells as *in vitro* model, increased translocation of HMGB1 to the nucleus was reported, which decreased Nrf2 expression and its subsequent downstream targets (Wu et al., 2021). Collectively, these findings suggest that HMGB1 acts as a positive regulator of ferroptosis *via* Nfr2 signaling. Targeting HMGB1 and ferroptosis therefore holds potential for the development of novel therapeutic strategies for DN.

Renal fibrosis is an important pathological process that contributes to the progression of CKD (Efstratiadis et al., 2009). The role of ferroptosis was demonstrated in studies using doxorubicin-induced renal fibrosis models (Fang et al., 2019). More specifically, increased expression of renal prostaglandin-endoperoxide synthase (Ptgs2) as a putative marker of ferroptosis was observed in the kidneys. Other ferroptosis-related molecules, including GPXs, iron and lipid peroxides have also been demonstrated (Sponsel et al., 1996; Himmelfarb, 2005; Zhang et al., 2022), corroborating the involvement of ferroptosis in the pathogenesis of renal fibrosis. Transforming growth factor β1 (TGF-β1) was shown to be a key mediator of renal fibrosis (Ikeda et al., 2014). More specifically, *in vitro* studies using renal tubular cells showed that TGF- β1 treatment increases the expression of SLC7A11 and GPX4, an effect that could be reversed by treatment with Ferrostain-1 (Fer-1), a well-characterized inhibitor of ferroptosis (Kim et al., 2021). The potential role and mechanisms underlying tubular cell ferroptosis during kidney fibrosis were demonstrated in kidney biopsies from patients with CKD and mouse models of fibrotic kidney disease unilateral ureter obstruction (UUO) or ischemia/reperfusion injury (IRI) nephropathy. Downregulation of the ferroptosis-associated marker GPX4 and upregulation of 4-hydroxynonenal (4-HNE) were observed (Li et al., 2017; Zhou et al., 2022). Moreover, inhibitors of ferroptosis were protective against kidney fibrosis in patients with CKD.

Ferroptosis has also been linked with ADPKD, a disease caused by mutations in polycystin-1 and -2 (PKD1 and PKD2) leading to the growth of cysts in the kidneys (Raaij et al., 2018; Zhang et al., 2021). Zhang et al. (2021) showed for the first time that ferroptosis regulates ADPKD progression, representing a promising therapeutic target (Zhang et al., 2021). Low levels of cell death were observed in Pdk1 mutant mice as a result of ferroptosis as opposed to apoptosis. Pdk1 mutant cells and kidney tissues from Pdk1 mouse models also showed decreased GSH expression and increased Transferrin receptor 1 (Tfr1), divalent metal transporter 1 (DMT1) and HO-1 expression. This resulted in high iron levels, low GSH and GPX4 activity, increased lipid peroxidation and ultimately ferroptosis. Erastin and Fer-1 prevented disease progression in Pkd1 mutant mice and 4-HNE expression, a lipid peroxidation product that increases in abundance during ferroptotic processes, increased in Pdk1 mutant renal epithelial cells, which regulated their proliferation *via* activation of Akt, S6, Stat3, and Rb. Collectively, these studies demonstrate the critical role of ferroptosis in the regulation of CKD progression, highlighting its promise as a novel therapeutic strategy.

## Targeting ferroptosis in chronic kidney disease

A range of preclinical studies have shown the potential of inhibitors of ferroptosis to prevent kidney disease. These include Fer-1, Rosiglitazone, Deferasirox (DFX) and Deferoxamine mesylate (DFO), and are summarized in Table 2.

**TABLE 2:** Ferroptosis-targeting inhibitors in CKD.

Inhibitors	Effect	Experimental model	Mechanism and outcome	References
Fer-1	Prevents ROS formation and lipid peroxidation	*Pkd1* ^*RC/RC*^ mice	Inhibits cell proliferation mediated by the activation of Akt, S6, Stat3, and Rb signaling during ferroptotic process	Zhang et al. (2021)
		Diabetic db/db mice	Regulates iron metabolism and inhibits HIF-1α/HO-1	Linkermann et al. (2014)
		Diabetic mice	Reduces lipid peroxidation *via* the HIF-1α/HO-1 pathway	Wiggin et al. (2008)
		UUO or IRI mouse model	Decreased FN and α-SMA, inflammatory cell accumulation, MCP-1 secretion and kidney fibrosis	Li et al. (2017)
Rosiglitazone	ACSL4 inhibitor	STZ-induced diabetic mice	Reduction of ROS, inhibition of NF-κB and reduced MCP-1 expression	Li et al. (2022)
		STZ-induced diabetic DBA/2 J mice	Reduced oxidative stress and regulation by novel transcription factors described	Bao et al. (2007)
		STZ-induced diabetic mice and db/db mice	Improved kidney function, reduction of lipid peroxidation and iron content	Wang et al. (2020)
DFO	Iron chelators	UUO or IRI mouse model	Reduction of FN and α-SMA, inflammatory cell accumulation and kidney fibrosis	Li et al. (2017)
		5/6 nephrectomy-induced CKD	Lower levels of renal injury and fibrosis *via* regulation of iron metabolism and the TGF-β1/Smad3 axis	Feng et al. (2021)
		UUO mouse model	Reduces renal iron accumulation by regulating TGF-β1-Smad3 and, oxidative stress signaling pathways	Zhou et al. (2022)
DFX		5/6 nephrectomy-induced CKD		Wang et al. (2022)
Tectorigenin	Antioxidant activity	UUO mouse model	Inhibits Smad3-mediated ferroptosis and fibrosis	Bebber et al. (2020)

Fer-1, Ferrostain-1; DFX, deferasirox; DFO, deferoxamine mesylate; Pkd1, Polycystin one; HIF-1α, Hypoxia-inducible factor 1-alpha; HO-1, Heme oxygenase-1; UUO, unilateral ureter obstruction; IRI, Ischemia/reperfusion injury; FN, fibronectin; α-SMA, alpha smooth muscle actin; MCP-1, Monocyte Chemoattractant Protein-1; STZ, streptozotocin; ROS, reactive oxygen species; ACSL4, Acyl-CoA, Synthetase Long Chain Family Member 4.

Under physiological conditions, iron homeostasis is maintained by hepcidin and iron regulatory proteins (Ueda et al., 1996). In CKD patients, iron homeostasis is disrupted as a result of altered iron uptake and/or insufficient iron export (Ueda et al., 1996). This leads to increased Fenton-mediated oxidative damage and renal injury (Naito et al., 2015). This suggests that iron accumulation in CKD patients occurs during ferroptosis. Therapeutic strategies to regulate the expression of iron metabolism-related proteins and alleviate ferroptosis through iron chelation (e.g., DFX or DFO) represent attractive approaches for the treatment of CKD. Indeed, studies (Table 2) using UUO or 5/6 nephrectomy-induced CKD rat/mouse models showed that DFX (Wang et al., 2022) and DFO (Li et al., 2017; Feng et al., 2021; Zhou et al., 2022) treatment reduced iron accumulation and renal injury through the regulation of the TGF-1β/Smad3 axis, inflammation and oxidative stress signaling pathways. As an alternative to iron chelators, the potential of Fer-1 to prevent ROS production and lipid peroxidation *via* hypoxia-inducible factor 1-alpha (HIF-1α)/HO-1 signaling was demonstrated in diabetic mouse models (Wiggin et al., 2008; Linkermann et al., 2014). In *Pdk1* mutant mice, Fer-1 could inhibit ferroptotic cell death, Akt-mediated proliferation, S6, Stat3, and Rb signaling, delaying cyst growth in ADPKD mouse models (Zhang et al., 2021). Treatment of STZ-induced DN animal models with rosiglitazone (an ACSL4 inhibitor), led to a reduction in lipid peroxidation and iron content in the kidneys, improving kidney function (Bao et al., 2007; Wang et al., 2020; Li et al., 2022). In more recent studies, tectorigenin showed protective effects against kidney injury and fibrosis, two key factors during CKD pathogenesis (Bebber et al., 2020). *In vitro*, tectorigenin suppressed ferroptosis and TGF-β1-stimulated fibrosis in primary renal tubular epithelial cells (TECs). Consistent with these studies, UUO-mouse models administered tectorigenin showed attenuated tubular cell damage and fewer fibrotic lesions in the kidneys as a result of the inhibition of Smad3-mediated ferroptosis and fibrosis. More specifically, tectorigenin inhibited Smad3 phosphorylation and the expression of Nox4, a downstream modulator of ferroptosis. Moreover, isoflavone could indirectly restore the expression of GPX4, a negative regulator of ferroptosis. Collectively, these studies highlight the potential of tectorigenin as a therapeutic strategy for CKD. Future studies are now required to dissect the mechanisms of action and potential therapeutic applications of this compound.

From a therapeutic standpoint, pharmacological modulators of ferroptosis have been explored *in vitro* and *in vivo* CKD experiments (Table 2). Despite promising results, the efficacy of these modulators and their potential side effects requires assessment in future clinical studies.

## Challenges and future perspectives

Numerous lines of evidence now support a role for ferroptosis in CKD, which is mediated by the iron-dependent accumulation of lipid peroxidation. Accordingly, we have discussed the major signaling pathways that regulate ferroptosis, its regulatory genes, the role of ferroptosis in CKD and potential therapeutic strategies to inhibit ferroptosis. Despite obvious progress, future studies must address the following concerns:

1) Ferroptosis is not an isolated event and is closely associated with other forms of cell death (apoptosis, necrosis and autophagy). The molecular mechanism(s) regulating this cross-talk require characterization to reveal potential antagonistic or synergistic roles in the context of kidney disease, and more specifically CKD.
2) Although a range of regulatory factors of ferroptosis have been described, they do not represent specific markers of ferroptosis. In this regard, reliable biomarkers, specifically in the context of kidney disease remains a challenge.
3) Accumulating evidence indicates that ferroptotic cell death can inhibit tumor growth and improve the efficacy of chemotherapeutic drugs. ^[99]^ However, in kidney disease, ferroptosis may negatively impact kidney health. Further mechanistic studies are therefore warranted.
4) Although the link between ferroptosis and acute kidney injury (AKI) has been widely explored and characterized, studies on ferroptosis and CKD remain limited. Indeed, the majority of studies to-date have used *in vitro* or *in vivo* models. However, as *in vitro* cell culture conditions and animal models of kidney diseases drastically differ, the interpretation and establishment of a link between ferroptosis and CKD can be challenging. Methods to translate scientific research into clinical applications should therefore be developed.
